# Transforming an embodied conversational agent into an efficient talking head: from keyframe-based animation to multimodal concatenation synthesis

**DOI:** 10.1186/s40469-015-0007-8

**Published:** 2015-09-08

**Authors:** Guillaume Gibert, Kirk N. Olsen, Yvonne Leung, Catherine J. Stevens

**Affiliations:** 1 The MARCS Institute, University of Western Sydney, Locked Bag 1797, Penrith, NSW 2751 Australia; 2 INSERM U846, 18 avenue Doyen Lépine, 69500 Bron, France; 3 Stem Cell and Brain Research Institute, 69500 Bron, France; 4 Université de Lyon, Université Lyon 1, 69003 Lyon, France

**Keywords:** Embodied conversational agent, Facial animation, Talking head, Motion capture, Multimodal speech synthesis

## Abstract

**Background:**

Virtual humans have become part of our everyday life (movies, internet, and computer games). Even though they are becoming more and more realistic, their speech capabilities are, most of the time, limited and not coherent and/or not synchronous with the corresponding acoustic signal.

**Methods:**

We describe a method to convert a virtual human avatar (animated through key frames and interpolation) into a more naturalistic talking head. In fact, speech articulation cannot be accurately replicated using interpolation between key frames and talking heads with good speech capabilities are derived from real speech production data. Motion capture data are commonly used to provide accurate facial motion for visible speech articulators (jaw and lips) synchronous with acoustics. To access tongue trajectories (partially occluded speech articulator), electromagnetic articulography (EMA) is often used. We recorded a large database of phonetically-balanced English sentences with synchronous EMA, motion capture data, and acoustics. An articulatory model was computed on this database to recover missing data and to provide ‘normalized’ animation (i.e., articulatory) parameters. In addition, semi-automatic segmentation was performed on the acoustic stream. A dictionary of multimodal Australian English diphones was created. It is composed of the variation of the articulatory parameters between all the successive stable allophones.

**Results:**

The avatar’s facial key frames were converted into articulatory parameters steering its speech articulators (jaw, lips and tongue). The speech production database was used to drive the Embodied Conversational Agent (ECA) and to enhance its speech capabilities. A Text-To-Auditory Visual Speech synthesizer was created based on the MaryTTS software and on the diphone dictionary derived from the speech production database.

**Conclusions:**

We describe a method to transform an ECA with generic tongue model and animation by key frames into a talking head that displays naturalistic tongue, jaw and lip motions. Thanks to a multimodal speech production database, a Text-To-Auditory Visual Speech synthesizer drives the ECA’s facial movements enhancing its speech capabilities.

**Electronic supplementary material:**

The online version of this article (doi:10.1186/s40469-015-0007-8) contains supplementary material, which is available to authorized users.

## Background

Embodied Conversational Agents (ECAs) can use verbal and nonverbal channels of communication to interact with human partners. On the one hand and in the ECA research community, a large amount of work has been devoted to improve ECAs’ capabilities to communicate by implementing human-like facial expressions, body gesture, and body posture (Pelachaud 2009), but relatively little research has focused on speech capacities in ECAs. Therefore, ECAs have strong dialog capabilities but weak speech production capabilities (Gris et al. 2014). In general, ECA animation is driven using MPEG-4 Facial Animation Parameters (FAPs). Unfortunately, most FAPs are low-level parameters that do not take into account speech specific gestures (Bailly et al. 2003). On the other hand, the speech research community has focused on visual speech capacities by developing dedicated virtual agents called talking heads. Early talking heads were based on simple animation techniques using a set of key frames (most of the time, visemes (Fisher 1968); i.e., a visually distinguishable face/mouth shape unit) coupled with a set of rules to create the correct transitions between those key frames (Cohen and Massaro 1993). However, this approach is not sufficient to create high quality auditory-visual (AV) speech synthesis. Research has now progressed with new animation techniques using corpora of multimodal speech uttered by humans. The corpora may be based on videos, such as those used in (Ezzat and Poggio 2000; Cosatto and Graf 2000). These approaches have created high quality transitions between visemes that have resulted in impressive synthetic AV speech. In fact, naive subjects are not able to distinguish real from synthetic stimuli (Turing test) (Ezzat et al. 2002). Nevertheless, these systems fail to increase intelligibility when compared to audio-only stimuli (Geiger et al. 2003). Furthermore, some synthesizers need 3D data in order to capture speech articulation. Photogrammetric recordings with beans glued to a speaker’s face can provide high resolution facial movement data (Bailly et al. 2002). This passive sensor approach necessitates a tedious preprocessing phase to construct a model that is able to fit unseen data. Other recording techniques have used active sensors to retrieve the positions of sensors over time without necessitating preprocessing. Kuratate (Kuratate 2008) used an Optotrak device to record a speaker’s facial movement while uttering speech and created a high quality system able to synthesize AV speech from any text input. Other active sensor equipment has been used such as Electro-Magnetic Articulograph (EMA) to record three dimensional speech articulation database to drive data-driven talking heads (Sheng et al. 2011) for computer assisted pronunciation training.

Humans commonly employ speech reading in adverse listening conditions to facilitate speech perception (Sumby and Pollack 1954). The ability to visually obtain phonetic information depends on watching facial movements that are produced by the speech articulators: mainly by the lips and jaw, and to some extent the larynx and tongue. These movements have been shown to be highly correlated with speech acoustics (Yehia et al. 2002). Although the tongue is a partly occluded speech articulator, its movements provide useful information for visual speech perception. Perceivers perform better with point-light displays including additional dots on the tongue and the teeth than with displays with ‘lips only’ dots during speech perception experiments (Rosenblum et al. 1996). Accurate 3D tongue models have been included in talking heads. These models are usually obtained by Magnetic Resonance Imaging (Badin et al. 2002; Engwall 2000; Badin et al. 2008) and animated by electromagnetic articulography data (Engwall 2003; Gibert et al. 2012; Steiner et al. 2013) or ultrasound images (Fabre et al. 2014). Computational approaches such as convolutive Nonnegative Matrix Factorization could be used to derive interpretable movement primitives from speech production data (Ramanarayanan et al. 2013). These speech movement primitives can be used to animate virtual agents’ speech articulators for a given set of activation data. EMA may be also used to synthesize acoustic speech from the variation of articulatory parameters (Toutios et al. 2013). A promising next step is to use synchronous recordings from EMA and motion capture data systems (Jiang et al. 2002; Engwall 2005). With such a setup, a large number of sensors can be placed on the speaker’s face and tongue.

In the present paper, we propose an innovative method to transform an existing ECA animated by interpolation between key frames (i.e., with poor speech capabilities) into a talking head. First, we describe the recording and processing of a multimodal synchronous speech database. An Optotrak Certus (Northern Digital Inc.) motion capture system and a Wave (Northern Digital Inc.) electromagnetic articulography system were used to record an Australian speaker uttering a large set of phonetically-balanced sentences. This unique setup enabled lip, jaw, and tongue trajectories to be recorded synchronously. We built an articulatory model by decomposing each speech articulator movements separately using guided Principal Component Analysis (gPCA). Sensor positions were converted into values of articulatory parameters in order to be used to control most ECAs. Second, the ECA’s original animation was modified: face and tongue key frames were transformed into articulatory parameters. Finally, we used the multimodal database to animate the ECA and used the MaryTTS software (Schröder et al. 2011) to create a multimodal text-to-speech synthesizer. This innovative approach can be applied to most ECAs (whose animation module is open) to improve their speech capabilities.

## Multimodal database

### Method

#### Setup

**Fig. 1:**
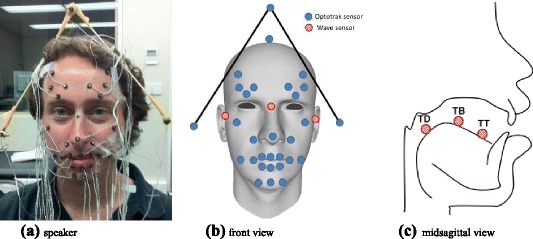
Sensor positions on the speaker’s face and tongue. A headset with 4 sensors was used to estimate the rigid head motion. The Wave sensors (in red) were glued to the tongue tip, tongue body and tongue dorsum and attached to the nasion and tragus. The Optotrak sensors (in blue) were attached to the face and the headset

An EMA system (Wave, Northern Digital Inc.) and an active motion capture (mocap) system (Optotrak Certus, Northern Digital Inc.) were used to record the position of sensors attached to the face and the tongue during a speech production session. These two systems were manufactured to record synchronously the position of their respective sensors together with the acoustic signal. There were 30 mocap active sensors attached to the speaker’s face: 3 for jaw motion, 8 for lip motion, 6 for eyebrow motion and 4 for rigid head motion mounted on a headset. Four additional sensors were attached to the Wave transmitter to align the Optotrak and the Wave referential systems. There were also 6 EMA sensors: 3 glued (using dental glue) on the tongue (tongue tip – **TT**, tongue body – **TB**, and tongue dorsum **TD**) and 1 attached to the nasion and 2 to the tragus. The positions of the sensors on the speaker’s face are displayed in Fig. 1.

The EMA field transmitter emits an electromagnetic field and signals transduced in small sensors within the field are resolved into spatial positions. The optimal measurements were within a 30 cm virtual cube oriented to the transmitter unit. The system delivered three spatial (x,y,z) measurements per sample and per sensor at 100 Hz. The accuracy of the tracking system has been previously assessed and validated for speech research (Berry 2011).

In the study, the positions of 34 Optotrak sensors were recorded at 60 Hz. The positions of the 6 Wave sensors were recorded at 100 Hz. The frames were time-stamped with respect to the beginning of the Optotrak recordings. The audio signal (mono, 22.05 kHz, 16 bits) was recorded synchronously by the EMA system.

#### Participant

A 30-year old male native speaker of Australian English participated in this recording.

#### Design and procedure

The speaker was seated close to the Wave transmitter and facing the Optotrak device. The phonetically-balanced sentences of the Lips challenge (Theobald et al. 2008; Theobald 2003) were pronounced by an experimenter and displayed on a screen facing the speaker. The speaker was instructed to repeat each sentence in a neutral tone after the experimenter. A set of 278 phonetically-balanced sentences was recorded. For each sentence a recording session of 10 s was set. Therefore, the total number of frames (at 60 Hz) was 166800.

### Data modeling

The recorded data are multimodal in essence: face, tongue trajectories and acoustic signal. Several processing steps were necessary before using the database to animate the ECA. In fact, sensor trajectories cannot be used directly to animate the ECA because of affordance issues. The shape model of an ECA may significantly differ from human morphology. The articulatory modelling provided ‘normalized’ animation parameters that can be used to control the ECA even if the shape model is different from the speaker’s morphology. These processes will be explained in the following subsections.

#### Acoustic segmentation

The audio files were automatically segmented using the method exposed in the MaryTTS (http://mary.dfki.de/) import voice procedure (Pammi et al. 2010). EHMM acoustic labeler from festvox (Black and Lenzo 2007) was used to generate label files from the audio files and corresponding transcriptions. The label files created by this procedure used the SAMPA phonetic alphabet and were stored as lab files. These files were then converted into Textgrid files by a custom-made Matlab (MathWorks, Inc., Natick, Massachusetts, United States) program. The software Praat (Boersma and Weenink 2010) was used to manually check and correct the segmentation. Therefore, for each audio file, a corresponding text file listed the series of phonemes and their timing information. From these text files, other segmentation files were created containing the series of diphones (i.e., the part of speech comprised between successive stable allophones) and their timing information. These files were used to build the multimodal dictionary that was then used to generate synthetic multimodal speech.

#### Articulatory model

The Optotrak and Wave devices recorded at different sampling rates. The Wave data were time-stamped by the Optotrak device with respect to the beginning of the Optotrak recording. The Wave data were downsampled (low-pass filtered at 20 Hz) to 60 Hz and, if acquisition timing differed between the Wave and the Optotrak devices, interpolated between two consecutive frames. After this step, the Wave and Optotrak data were sampled at 60 Hz and synchronized.

Head movement (translations and rotations) was estimated and corrected using the Optotrak sensors positioned on the headset and the Wave sensors placed on the nasion and the tragus. Speech articulator movements did not affect these sensors. A modeling procedure was applied to the data with two specific aims: first, to extract meaningful parameters controlling an elementary articulator, and second, to remove artifacts and measure noise. PCA was applied to rigid motion. The first two components explained more than 90 % of the total variance. An articulatory model was built using the method proposed by (Gibert et al. 2005; Revéret et al. 2000; Badin et al. 2002). A pruning step (simple vector quantization) was applied to remove the frames in which sensor positions were too similar (Euclidian distance < 1.0 mm). This step conditioned the data before building the statistical models. Then, the contribution of the different speech articulators (jaw, tongue and lips) and the eyebrows was iteratively subtracted. This subtraction consisted of an iterative application of PCA on subsets of landmarks.

**Fig. 2:**
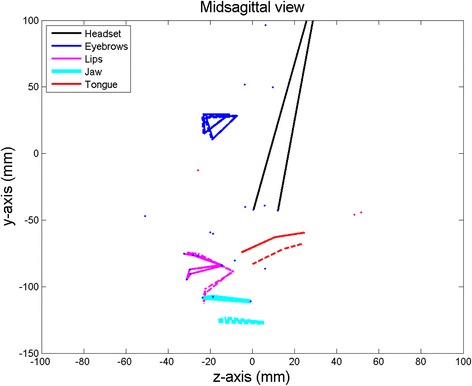
Maximum variations (solid and dashed lines) of the first articulatory parameter jaw1 driving the jaw. It displays the peaks of opening-closing movement. The tongue follows the jaw opening movement and this movement is encompassed by this articulatory parameter

**Fig. 3:**
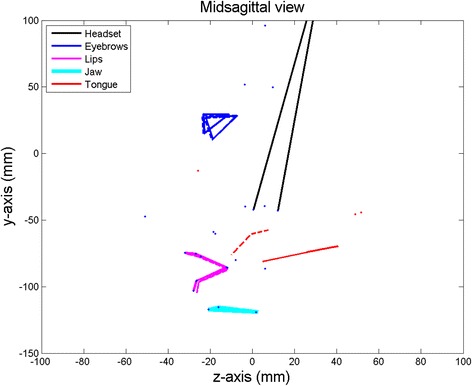
Maximum variations (solid and dashed lines) of the first articulatory parameter driving the tongue. The parameter tongue1 corresponds to the posterior and anterior extents of a tongue front-back movement

The procedure extracted 10 articulatory parameters; extreme variations of jaw1 and tongue1 are shown in Figs. 2 and 3 respectively:

- Jaw opening (**jaw1**) using PCA on the jaw position sensor values (13.40 % of the global variance);
- Tongue front-back movement (**tongue1**) using PCA on the residual tongue (*TB, TD*) position values (13.17 % of the global variance);
- Tongue flattening-bunching movement (**tongue2**) using PCA on the residual tongue (*TB, TD*) position values (4.83 % of the global variance);
- Tongue tip vertical movement (**tongue3**) using PCA on the residual tongue (*TT*) position values (5.13 % of the global variance);
- Tongue tip horizontal movement (**tongue4**) using PCA on the residual tongue (*TT*) position values (5.69 % of the global variance);
- Lip rounding (**lips1**) using PCA on the residual lip position values (10.06 % of the global variance);
- Lip closing (**lips2**) using PCA on the residual lower lip position values (0.93 % of the global variance);
- Lip raising (**lips3**) using PCA on the residual upper lip position values (2.66 % of the global variance);
- Jaw rotation (**jaw2**) using PCA on the residual jaw position sensor values (2.04 % of the global variance);
- Eyebrow movements (**eyebrows1**) using PCA on the residual eyebrow position values (4.23 % of the global variance).

The maximum variations of the first articulatory parameter driving the jaw (**jaw1**) opening-closing movement are shown in Fig. 2. The tongue is carried by the jaw and this articulatory parameter also drives a tongue rotation around a point at the back of the tongue (Badin and Serrurier 2006). Four additional parameters driving the tongue movements were derived from the data. Figure 3 represents the maximum variations of the first of them. The parameters **tongue1** and **tongue2** were extracted using the position of TB and TD sensors only. They controlled the front-back and flattening-bunching movements. The parameter **tongue3** was extracted by guided PCA using the position of TT sensor only.

Three articulatory parameters driving the lips were extracted. They corresponded to the lip protrusion (**lips1**), lip closing (**lip2**) and lip raising (**lip3**) movements. Finally, a parameter (**eyebrows1**) driving the eyebrow movements was extracted as it may convey prosodic information (Granstrom and House 2005).

### Data reconstruction

The rigid head motion was estimated in terms of translations (Tx, Ty, Tz) and rotations (Rx, Ry, Rz) around the neck (the center of rotation was estimated at the same time). These movements were transformed into articulatory parameters using the following equations:

$$ \hat{\alpha_H}=\underset{\alpha_H\in {\left[-3;3\right]}^N}{\mathrm{argmin}}\parallel {\hat{P3D}}_{HEADSET}-P3{D}_{HEADSET}{\parallel}_2 $$

$$ {\hat{P3D}}_{HEADSET}= RigidMotion\left({m}_{HEADSET,\ }\ {m}_{Hmvt}+{\alpha}_H\ eig{v}_H\right) $$

where ***P***3***D***_***HEADSET***_ corresponded to the actual position of Optotrak sensors placed on the headset, and ${\hat{\boldsymbol{P}3\boldsymbol{D}}}_{\boldsymbol{HEADSET}}$ corresponded to the estimated ones, ***m***_***HEADSET***_ corresponded to the average position of the sensors on the headset, ***m***_***Hmvt***_ corresponded to the mean rigid head motion of the model, ***α***_***H***_ corresponded to the rigid motion parameter values to be estimated and ***eigv***_***H***_ corresponded to the rigid motion model derived from PCA. The number ***N*** of rigid motion parameters driving the headset was 6. The values of each parameter were limited to [−3; 3].

Each recording was then ‘inversed’, i.e., for each frame of the recording, the values of the articulatory parameters were estimated to minimize the Euclidian distance between the original data and the reconstructed ones using the following equation:

$$ \hat{\alpha}=\underset{\alpha \in {\left[-3;3\right]}^M}{\mathrm{argmin}}\parallel {m}_{Face}+\alpha\ eig{v}_{Face}-P3{D}_{WAVEOPTO}{\parallel}_2 $$

where ***P***3***D***_***WAVEOPTO***_ corresponded to the position of the Wave and Optotrak sensors after subtraction of the rigid head motion, ***m***_***Face***_ corresponded to the mean face configuration of the articulatory model, ***α*** corresponded to the articulatory parameter values and ***eigv***_***Face***_ corresponded to the articulatory model. The number ***M*** of articulatory parameters driving the tongue was 10. The values of each parameter were limited to [−3; 3]. This inversion used the relation between the relative sensor positions to recover the position of missing data. Therefore, the sensor trajectories were transformed into variations of articulatory parameter values.

**Fig. 4:**
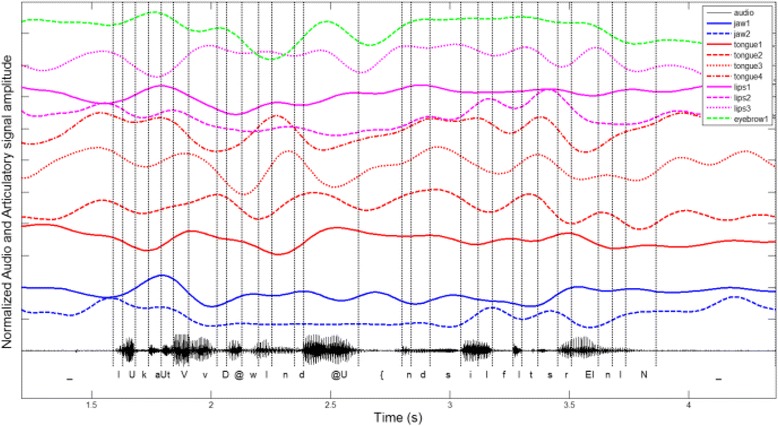
Variation of the ten articulatory parameter values for the sentence: “Look at the windows and see if it’s raining”. The variations are nonlinear and cannot be replicated with linear interpolation animation. The database is segmented into phonemes. A multimodal diphone dictionary is created with this segmented database

A low-pass Butterworth filter (6^th^ order, 8Hz) was applied to the articulatory parameters in order to remove noise due to recordings and missing data. An example of variation of the articulatory parameter values across time for a sentence of the corpus can be seen in Fig. 4. The variation of these parameters is clearly nonlinear. Animation approaches that use linear interpolation between key frames cannot replicate such variations. After this filtering, the recordings were reconstructed, i.e., missing data were estimated using the articulatory model. The reconstruction error computed as the Euclidian distance between the recorded position of the Optotrak/Wave sensors and the reconstructed ones was M = 5.41 mm and SD = 3.15 mm. An example of data reconstruction can be found in the Additional file 1: Video S1. This dataset comprised a large amount of English phonemes in different contexts. A multimodal diphone dictionary was created. It contained for each diphone, the variations of the articulatory parameters driving the visible and partly occluded speech effectors.

## Embodied Conversational Agent

### Avatar

The avatar used in this study was a representation of an Australian performance artist, Stelarc. This 3D model was originally driven by a set of key frames controlling the visible and partially occluded speech facial articulators such as lips, jaw, and tongue. The full animation was originally created by linear interpolations between those key frames. Unfortunately, linear interpolations do not accurately replicate speech articulator movements. This is one of the reasons why we developed a new animation method.

### Face articulatory parameters

Selected key frames (from the original model) were used to create articulatory parameters for driving the avatar. The vertex coordinates of the neutral pose were subtracted from the vertex coordinates of each key frame. The resulting variation between these positions was then variance-normalized and set to vary between 0 and +3. Synthetic articulatory parameters controlling the jaw (and the mandible) (**jaw1**) and the lips (**lips1**, **lips2**, **and lips3**) were created. These parameters corresponded to the facial articulatory parameters derived from EMA data. Note that no parameter corresponding to **jaw2** was found in the available key frames. This parameter recovered 2 % of the global variance in the EMA data. It was not included in the final set of synthetic articulatory parameters for the animation.

### Tongue articulatory parameters

**Fig. 5:**
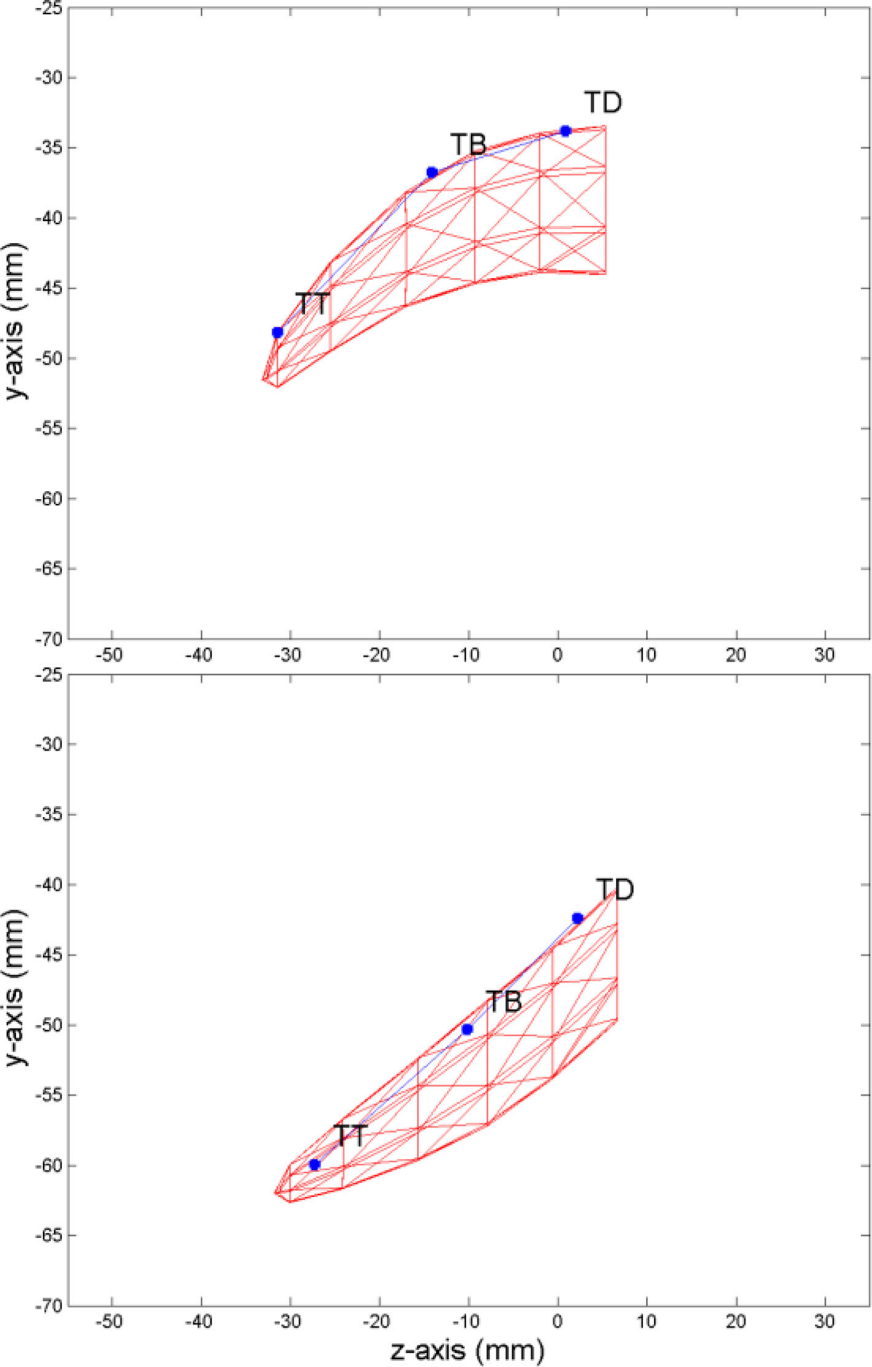
Two tongue configurations (midsagittal view) from the quantized EMA database (TD, TB and TT sensor positions in blue) and the corresponding constrained 3D tongue mesh (red mesh)

Because tongue key frames were not related to any speech articulation in the original animation, but only to meaningless geometric variations, an alternative method was designed. Each tongue sensor from EMA data was associated with a specific vertex of the 3D tongue mesh (which is composed of 50 vertices) of the original ECA face model. For each sample of the quantized EMA database, tongue postures were determined by estimating the best linear mixture of weighted key frames that minimized the distance between the EMA tongue sensor positions and the corresponding tongue mesh vertices. The least square estimation of the vector of weights α was simply performed by:

$$ \hat{\alpha}=\underset{\alpha \in {\left[-10;10\right]}^N}{\mathrm{argmin}}\parallel \sum_i^N{\alpha}_iP3{D}_{K_i}-P3{D}_{EMA}{\parallel}_2 $$

where $\boldsymbol{P}3{\boldsymbol{D}}_{{\boldsymbol{K}}_{\boldsymbol{i}}}$ corresponded to the position of the three selected vertices of the 3D tongue mesh for the key frame ***K***_***i***_, ***α***_***i***_ corresponded to the weights applied to the key frame ***K***_***i***_, and ***P***3***D***_***EMA***_ corresponded to the position of the three EMA sensors TD, TB and TT. The number of key frames available in the original model was N = 9. The values of each weight ***α***_***i***_ were limited to [−10; 10]. Examples of configurations found in the EMA database and the corresponding constrained 3D tongue mesh are visualized in Fig. 5.

After this step, a quantized database of 3D tongue postures was created. For all the configurations of the EMA database, corresponding constrained 3D tongue mesh of 50 vertices was available. The reconstruction error computed as the Euclidian distance between the EMA sensor (TD, TB and TT) positions and the specific vertices of the 3D tongue mesh was M = 7.07 mm and SD = 6.94 mm.

**Fig. 6:**
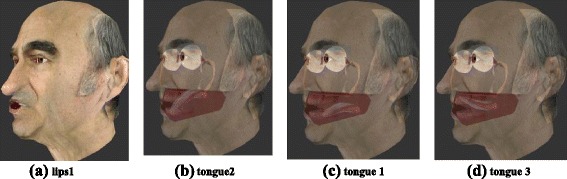
Examples of the maximum variation (one direction) of some articulatory parameters driving the avatar. **a lips1** corresponds to lip protrusion; **b tongue2** corresponds to the flattening-bunching movement, **c tongue1** corresponds to the posterior extent of a tongue front-back movement, **d tongue3** corresponds to the peak of a tongue tip vertical movement

The same procedure as described in section 4.2.2 was used to build a tongue articulatory model using the database of 3D tongue postures in addition to the EMA database. Finally, the articulatory tongue model was controlled by 5 articulatory parameters (as described in (Badin and Serrurier 2006)): jaw height/opening (**jaw1**), tongue front-back (**tongue1**), tongue flattening-bunching (**tongue2**), tongue tip vertical (**tongue3**) and tongue tip horizontal (**tongue4**). Examples of the maximum variation of key articulatory parameters are shown in Fig. 6. This avatar was driven by similar articulatory parameters to the ones derived in the modelling procedure described above (Gibert et al. 2012).

## Auditory-Visual Text-To-Speech system

### Overview

**Fig. 7:**
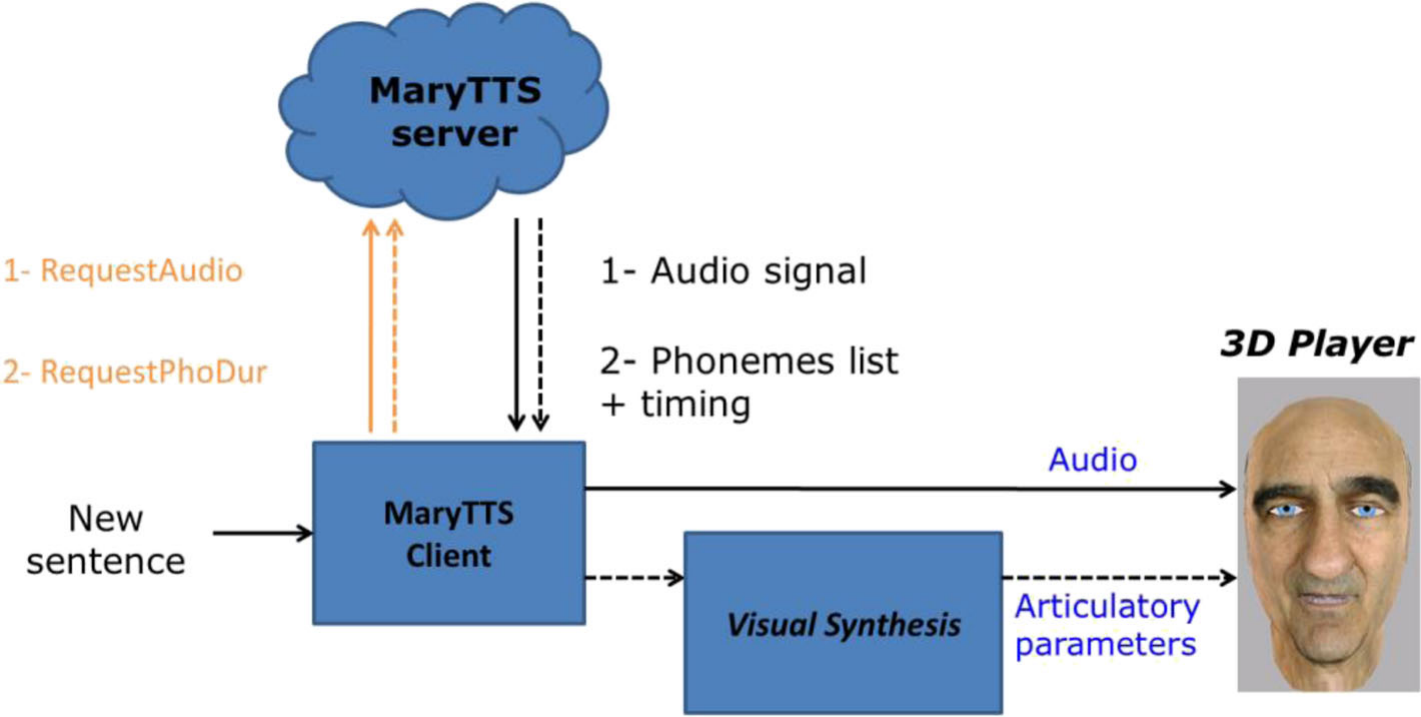
Schematic representation of the auditory-visual speech synthesis system. Given a new sentence to pronounce, the program acts as a MaryTTS client and asks for an audio signal corresponding to this sentence and to the list of phonemes with their duration (provided by the prosodic module embedded in the MaryTTS software). From the list of phonemes, a second program performs the visual synthesis. It searches the best series of diphones given selection and concatenation costs in the multimodal dictionary. This series is processed to match the expected duration and minimizes the gaps at each boundary. Finally, the acoustic signal and the variation of the articulatory parameters are passed to the 3D Player which animates the ECA accordingly

The system proceeds in three steps. First, a module connects to the MaryTTS server (http://mary.dfki.de/) and asks for the list of phonemes and its duration for a given text. Second, the same module requests an acoustic signal corresponding to the given text. Third, the list of phonemes plus duration is sent to the Visual Text-To-Speech (VTTS) module, which searches the best list of diphone candidates and concatenates them to create articulatory parameter trajectories. The acoustic signal and the articulatory parameter trajectories are sent to the animation module, which plays the data i.e., the ECA speaks and moves his speech effectors (jaw, lips and tongue) accordingly. The schematic representation of the system is shown in Fig. 7.

### Text-to-Speech

The first step of our system is to ask the MaryTTS server (Schröder et al. 2011) to generate an acoustic signal that corresponds to the new sentence. To this end, the MaryTTS server performs natural language processing to generate a list of phonemes and prosodic information (duration, pitch variation, intensity). Finally, the synthesizer creates a sound signal from this information using a cluster unit selection code derived from FreeTTS. In fact, the recorded speech signal was used to create a specific MaryTTS synthetic voice for our speaker following the procedure described in the voice creation module (Pammi et al. 2010). This procedure performs feature extraction from acoustic and text data and then automatic segmentation/labelling. The procedure was bypassed at this stage to check manually the automatic segmentation/labelling (see subsection 4.2.1). Then, the system builds a unit selection voice from the manually checked segmentation files and the acoustic features. Given the manual segmentation, the quality of the voice is better than the fully automatic procedure that can generate artifacts because of segmentation errors.

The second step consists of asking the MaryTTS server (Schröder et al. 2011) to provide the list of phonemes with their duration. This information is sent to the visual synthesis system. The generation of visual speech is explained in the following section.

### Visual synthesis

**Fig. 8:**
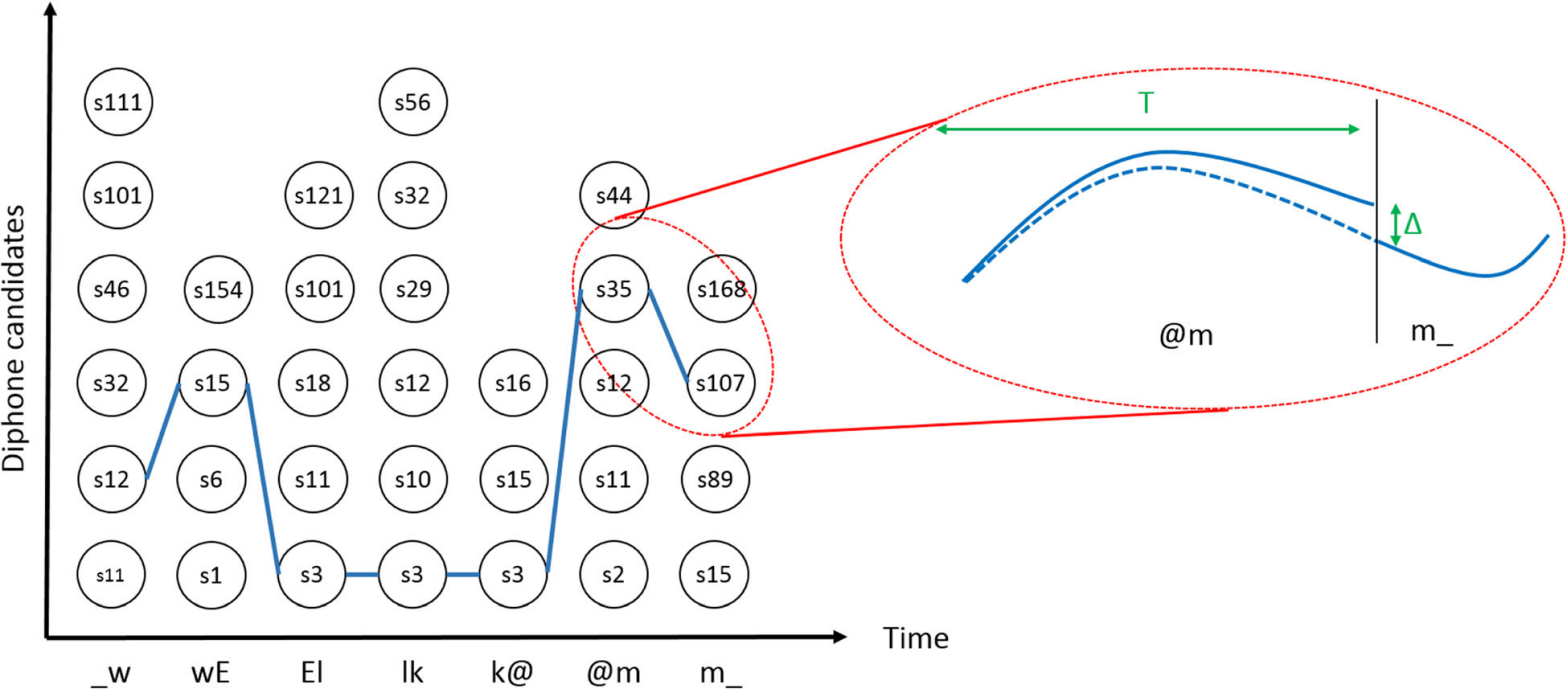
Schematic representation of the visual synthesis system. Given a series of diphones (e.g., “Welcome”), the system searches in the diphone dictionary the candidates and creates a trellis of diphones (i.e., a list of diphone candidates). The best series of diphones is selected using a concatenation cost, i.e., the aim is to minimize the RMS distance between values of the articulatory parameters of the last frame of the previous diphone and the first frame of the current one. The selected diphones are linked by a blue line in this example. If consecutive diphones come from the same sentence, there is no gap at the concatenation borders (e.g., this is the case for the diphones El, lk and k@ in this example). In other cases, there is a gap (Δ) at the concatenation that must be minimized. A gapless processing step is applied to each articulatory parameter. It consists of adding a small value (equal to Δ/T*frame_index, where Δ corresponds to the difference between the values of the articulatory parameter of the last frame of the current diphone and the first frame of the following one, T corresponds to the total number of frames of the current diphone and frame_index corresponds to the current frame number) at each time step to reduce the gap while keeping the temporal nonlinear variation of the parameter values

Given the list of phonemes and their duration, the visual synthesis system creates a list of diphones and their corresponding duration. For instance, if the word “Welcome” is submitted to the MaryTTS server, it will return the following list of phones: _ w E l k @ m _ with their respective timing (in seconds): 0.060, 0.125, 0.19, 0.29, 0.385, 0.51, 0.695. The corresponding list of diphones is then derived as follows: _w, wE, El, lk, k@, @m, m_. The system searches in the multimodal diphone dictionary containing the trajectories of the articulatory parameters the various candidates for each diphone. This step generates a trellis of diphones (see Fig. 8). The best series of diphones is then selected using a cost function based on a concatenation weight. This step selects the best series of diphones that minimize the Root Mean Square (RMS) distance between values of the articulatory parameters of the previous diphone and the current diphone.

Except in the case that a series of diphones corresponds to a series contained in the dictionary (for instance, the diphones El, lk and k@ comes from the same sentence in the example of Fig. 8), there is always a gap at each concatenation frontier (for example, between the diphone @m and m_ in the example of Fig. 8). This gap is reduced by applying a gapless processing step on the articulatory parameters within the preceding diphone gradually (Gibert et al. 2005). It consists of adding a small value (equal to Δ* frame_index/frame_total_number, where Δ corresponds to the distance (i.e., gap) between the end of the current diphone and the beginning of next one, frame_index corresponds to the time step and frame_total_number corresponds to the total number of time steps for the current diphone) to the variation of the articulatory parameters at each time step. Even if there is a concatenation gap between two consecutive diphones as shown in Fig. 8, this procedure cancels it while keeping the nonlinear variations of the articulatory parameters. This way, the final sample of the previous diphone coincides with the first sample of the current one.

### Animation

Acoustic and articulatory data are processed in parallel. The system requests the audio file and the phone list together with timing to the MaryTTS server. The VTTS module creates a file containing the variation of the articulatory parameters with the same timing used by MaryTTS to create the acoustic file (as described in the previous subsection). Once the acoustic signal and the matrix (articulatory parameter x time) of articulatory parameter variations are available, two threads are started: one playing the sound and another one playing the face gestures. The animation module is a custom written software developed using Java and Java3D/JoGL. The animation module plays congruent and synchronous articulatory and acoustic signals from the same Australian speaker. In fact, any existing English MaryTTS voices could be used together with the articulatory data we recorded. However, the visual signals may become incoherent with American or British English voices for instance. Moreover, synchronization may be also different in this case. In our system, the same segmentation files were used to create the acoustic and articulatory sound units. Therefore, this module plays the multimodal data synchronously. An example of animation can be found in the Additional file 1: Video S1.  

Even though the articulatory parameters driving the avatar and the ones derived from the speaker have the same topology (e.g., **jaw1** controlled in both cases the jaw opening/closing), it may happen that positive variation of **jaw1** corresponded to jaw opening for the avatar’s model and jaw closing for the speaker’s model. The sign attribution was determined manually.

## Conclusions & perspectives

A method to transform an avatar with generic tongue model and animation by key frames into a talking head that displays naturalistic tongue, jaw and lip motions was described. First, a multimodal speech database consisting of the recording of face and tongue movements during the production of a large number of sentences by an Australian speaker was created. This database was processed to create a dictionary of synchronous multimodal diphones. An articulatory model of the ECA was then created by transforming selected key frames into articulatory parameters for the jaw, lips and tongue. Real articulatory data together with the acoustic signal were used to steer the talking head using the MaryTTS software (Schröder et al. 2011) to generate the synthetic acoustic signal and the phoneme and prosodic information. The original ECA with good non-verbal capabilities (facial expressions, blinks, gestures, pupil dilation/constriction (Gibert and Stevens 2012), etc.) and poor speech capabilities was transformed. The ECA kept its nonverbal capabilities intact and significantly improved its speech capabilities. Importantly, this method is a bridge between the ECA and speech communities. More methods should be established to take advantages of the development of the two communities to create virtual agents able to interact naturally with human partners. Human communication is multimodal in essence: verbal, coverbal and nonverbal channels of communication are used during face-to-face communication. Each community has developed specific models for verbal, coverbal and nonverbal behaviors. Bridges such as the proposed method are vitally important for virtual humans to efficiently use all possible channels of communication.

The proposed method could be easily extended to other realistic auditory-visual animation methods based on concatenation (Musti et al. 2011) or Hidden Markov Models (Bailly et al. 2009) using the same unique multimodal database. Electromagnetic articulography provides only a spatially sparse representation of the tongue movements. Co-registration methods of EMA and real-time magnetic resonance imaging (rtMRI) data (Kim et al. 2014) provides richer spatio-temporal data to animate the tongue movements and create a better 3D tongue model. The proposed method could be applied on the USC-TIMIT (Narayanan et al. 2014) which is an extensive database of multimodal (EMA, rtMRI, acoustics) speech production. This work could be extended to emotional speech production which generates different articulation patterns for critical speech articulators compared to neutral speech production (Kim et al. 2015). An evaluation of the method will be performed in the future to assess the gain in intelligibility; for instance, through a speech in noise perception experiment (Gibert et al. 2013). Furthermore, the proposed method could be applied to modify existing avatars that are not able to produce correct speech movements. This would allow hearing impaired people (Gibert et al. 2005) and second language learners (Engwall 2008) to effectively utilize a larger number of virtual agent applications.

## Additional file

**Additional file 1: Video S1.** The video illustrates the articulatory parameters (jaw1, jaw2, tongue1, tongue2, tongue 3, tongue 4, lips1, lips 2, lips3, eyebrows1) derived from the speech production database and their effects on facial movements. Then, an example of data reconstruction is shown, this processing step allows to recover missing data. Finally, an example of ECA animation using real speech production data is played. (MP4 14967 kb)
